# Comparison of Secular Trends in Cervical Cancer Mortality in China and the United States: An Age-Period-Cohort Analysis

**DOI:** 10.3390/ijerph13111148

**Published:** 2016-11-17

**Authors:** Jinyao Wang, Zhiqiang Bai, Zhenkun Wang, Chuanhua Yu

**Affiliations:** 1 Department of Epidemiology and Biostatistics, School of Health Sciences, Wuhan University, 115 Donghu Road, Wuhan 430071, China; jinjinyao456@163.com (J.W.); wongzhenkun@gmail.com (Z.W.); 2 College of Life Science and Technology, Huazhong Agriculture University, Wuhan 430070, China; baizq1987@126.com; 3 Global Health Institute, Wuhan University, 115 Donghu Road, Wuhan 430071, China

**Keywords:** cervical cancer, mortality, age-period-cohort analysis

## Abstract

*Background*: As one of the most common cancers in the female population, cervical cancer has ranked as the second most incident gynecological cancer in recent years, trailing only breast cancer. We aimed to assess and compare the secular trends in cervical cancer mortality in China and the United States and analyze the independent effects of chronological age, time period and birth cohort using age-period-cohort (APC) analysis. *Methods*: We performed an age-period-cohort analysis using the intrinsic estimator method to estimate the independent effects of age, time period, and birth cohort on cervical cancer mortality. We collected mortality data for China and the United States from the WHO Mortality Database and China Health Statistical Yearbook database. *Results*: We examined the general trends in cervical mortality rates in China and the United States during the periods 1988–2012 and 1953–2012, respectively. The age-standardized mortality rates (ASMRs) for cervical cancer in urban China, rural China and the U.S. showed a general decreasing trend during the observation period, except for urban China, which experienced a significant increase beginning in 2002. The mortality rates for cervical cancer in the three areas showed a general increasing trend with age, regardless of the period effect. Period effects declined steadily in both rural China (from 0.19 to −0.26) and the U.S. (from −0.20 to −0.43); however, a slight increasing trend was identified (from −0.25 to 0.33) in urban China, which indicated that the risk of mortality increased with time. Cohort effects peaked in the cohort born in 1911–1915 in both rural China and urban China, declined consistently in the cohort born before 1950, and then decreased again in the cohort born after 1976–1980. The cohort effect in the U.S. peaked in the birth cohort born in 1876–1880, then leveled off and slightly decreased in younger generations. *Conclusions*: Our study showed that in general, cervical cancer mortality rates increased with age and decreased with birth cohort in the U.S., while the risk of mortality was highest in the cohort born during 1946–1975 in urban China. Additionally, the risk of mortality consistently increased with age in women younger than 64 years old in urban and rural China and began to decline in older groups. Although the age and cohort effects were relatively strong, the period effect may be the key factor affecting cervical cancer mortality trends, mainly reflecting the immediate effects of effective treatment and the implementation of screening.

## 1. Introduction

As one of the most common gynecological cancers, cervical cancer ranks as the second most prevalent malignancy after breast cancer and the third leading cause of cancer-related mortality (after lung and breast cancer) among women worldwide [1,2,3]. Although a decreasing trend in cervical cancer mortality associated with the implementation of various preventive measures and clinical methods has been observed in a number of developed countries over the last several decades, it remains an important cause of morbidity and mortality that threatens the health and lives of women worldwide [4,5]. Therefore, to better understand factors associated with different cervical cancer mortality trends and assess the effects of public health control policies in different areas, analyzing the secular cervical cancer mortality trends in association with the independent effects of age, period and birth cohort seemed particularly important [6].

It has been estimated that approximately 530,000 women were newly diagnosed with cervical cancer in 2008 and more than 250,000 women die from cervical cancer each year worldwide [7,8]. Of the cases of cervical cancer identified worldwide, nearly 78% occurred in developing countries while developed countries accounted for only 4.4% of the total cases [9]. In the United States—the largest developed country in the world, approximately 12,360 cervical cancer cases and more than 4000 cervical cancer deaths were reported in 2014 [10]. As the world’s largest developing country, it has been estimated that nearly 100,000 cervical cancer cases, accounting for approximately 20% of total new cases globally, and nearly 30,000 women cervical cancer deaths occur in China each year [7]. Due to widespread implementation of cytology screening since its introduction in the 1960s, cervical cancer mortality rates have declined in the United States and China; however, declines in the United States have recently leveled off, and the mortality rate in China remains high in some impoverished areas including Gansu, Shanxi and Western China [7,11]. Overall, the specific reasons for the aforementioned trends in cervical cancer mortality rates remain unclear, and there is an urgent need for more research to be conducted to explore the underlying causes of these trends.

The age-period-cohort (APC) model is a common statistical method that has been widely used to describe and explain secular trends in disease rates in populations over time, especially for cancer [12,13]. Age, period (year of death), and cohort (year of birth) are three independent factors that have been found to be associated with cancer mortality, and, therefore, each of these factors may affect trends in cancer mortality. The age effect represents the differential risks associated with membership in different age groups. The period effect represents the variation in risk associated with certain periods or years that effect all age groups. The cohort effect indicates the temporal changes associated with birth during the certain period, including socioeconomic factor, changes in lifestyle and exposure to risk factors that differ in different generations [14,15,16]. While traditional cohort studies utilize analytic techniques that fails to adjust for the period effect and cannot simultaneously consider the effects of age, period and cohort, the APC model can consider these three factors simultaneously. Therefore, the APC model was applied to separate the independent effects of chronological age, time period, and birth cohort on secular trends in cervical cancer mortality [14,17].

To the best of our knowledge, studies focusing on the comparison of cervical cancer mortality trends in China and the United States using APC analysis have been rarely conducted. Therefore, to better compare and assess the temporal trends in cervical cancer and the underlying reasons for these trends, we examined the longitudinal trends in cervical cancer mortality in China and the United States, during the periods 1988–2012 and 1953–2012, respectively. Furthermore, we used the APC model combined with the Intrinsic Estimator algorithm to analyze the independent effects of chronological age, time period and birth cohort.

## 2. Materials and Methods

### 2.1. Data Source

Cervical cancer mortality data were obtained from the WHO Mortality Database and the China Health Statistical Yearbook database for two countries: China and the United States. In our study, data for China were only available in the WHO Mortality Database from 1988 to 2000; therefore, data for the period 2002–2012 were collected from the China Health Statistical Yearbook database. Because mortality data for 2001 were not available, we calculated the average of the surrounding four calendar years instead of the average value for 1998–2002 [18]. In this study, data for China were obtained from urban and rural regions of mainland, the data of urban China include municipalities and prefecture-level cities, and the data of rural China include counties and county-level cities. The data of China in our study covered 31 provinces, cities and municipalities, accounting 1/100 of the total population. With reference to China, the data were collected from Vital Registration, the Ministry of Health, China Vital Statistics-Deaths, and the mortality data for China comes from 161 Death Cause Surveillance Points nationwide, which used multi-stage stratified randomized sampling. Therefore, the data are basically reliable. To better understand the temporal trends and accurately analyze the secular trends in urban and rural regions in mainland China, we collected data for urban and rural regions in China from 1988 to 2012. For comparison, we also collected cervical cancer mortality data for the United States during the period from 1953 to 2012 [19].

Cervical cancer was defined as the International Classification of Diseases of the 10th revision (ICD-10) code C53 [5]. During the period 1957–1977, four versions of the ICD were used to code for cervical cancer, and the cervical cancer code changed from 171 (ICD7) to C53 (ICD-10). Cervical cancer cases aged 20–79 years were included in this study; because patients with cervical cancer under the age of 20 years old are very rare, and patients over the age of 80 die from many other competing causes, cases below 20 years old and above 80 years old were excluded from our study [20]. To increase the comparability of the three regions, age-standardized rates were calculated adjusted to the world standard population using the direct method (1960), which was proposed by Segi [21] and modified by Doll et al. [22]. Cases of cervical cancer in urban and rural China were divided into twelve 5-year age groups (20–24, 25–29, …, 75–79) and five 5-year calendar periods (1988–1992, 1993–1997, …, 2008–2012). Similarly, cases in the United States were also divided into twelve 5-year age groups (20–24, 25–29, …, 75–79) and twelve 5-year calendar periods (1953–1957, 1958–1962 ,…, 2008–2012) [6].

### 2.2. Statistical Analysis

The age-period-cohort (APC) model has been widely used in the fields of sociology and epidemiology over the past 80 years [17,23,24]. The APC model is based on the Poisson distribution and can reflect temporal trends in diseases by age, period and cohort and after adjustment for age, period and cohort. However, a non-identification problem may still exist as there is a linear relationship between the age, period and cohort, which makes it difficult to estimate the unique set for every age, period and cohort effect [17]. To solve this problem, the intrinsic estimator (IE) method, which is associated with the APC model and was proposed by Yang and Fu [25], was used [17,26]. We used the Akaike information criterion (AIC) and the Bayesian information criterion (BIC) to evaluate the goodness-of-fit of the model. The STATA 12.0 software (StataCorp, College Station, TX, USA) and SAS 9.13 (SAS Institute, Cary, NC, USA) programs were chosen as the statistical analysis software for all statistical analyses.

## 3. Results

**Figure 1:**
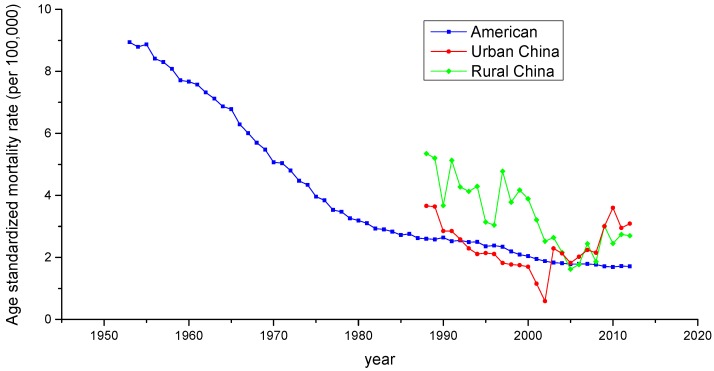
Trends of age-standardized mortality rates per 100,000 population for cervical cancer in China and the U.S.

Age-standardized mortality rates (ASMRs) for cervical cancer in China and the United States during the period 1953–2012 are shown in Figure 1. Appendix A Table A1, Table A2 and Table A3 show the age-specific mortality rates for cervical cancer by year of death in three regions; urban China, rural China and the United States. As is presented in Figure 1, the ASMRs for cervical cancer in the three regions generally showed a decreasing trend during the observation period, except for urban China, which experienced a significant increase beginning in 2002 and showed a pronounced V-shaped trend. Overall, rural China had the highest mortality rate during the period from 1988 to 2004, whereas the ASMR observed in urban China was higher than the other two countries during the period from 2005 to 2012. The ASMR in the U.S showed a steep decreasing slope during the period 1953–1982, but leveled off thereafter.

**Figure 2:**
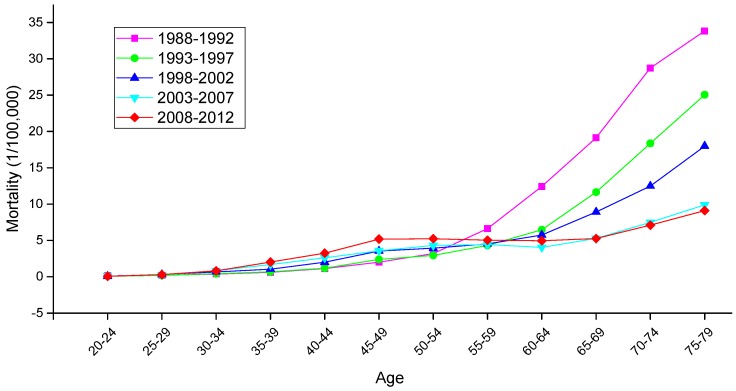
Age-specific mortality rates for cervical cancer per 100,000 female population in different periods in urban China.

**Figure 3:**
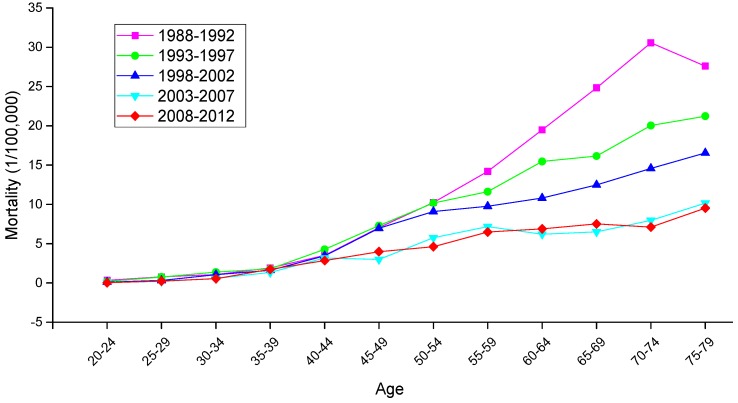
Age-specific mortality rates for cervical cancer per 100,000 female population in different periods in rural China.

**Figure 4:**
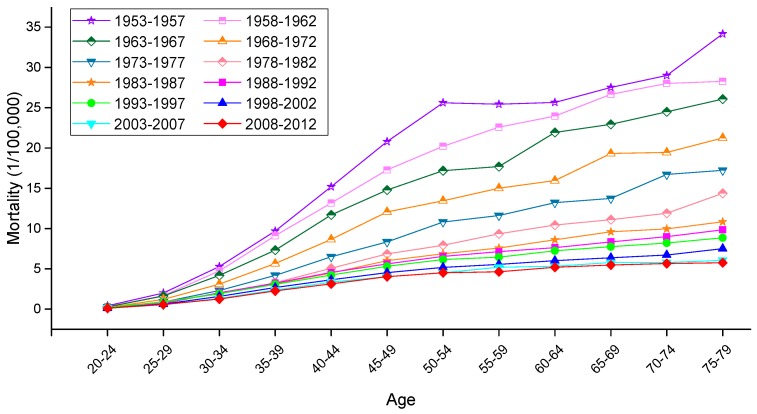
Age-specific mortality rates for cervical cancer per 100,000 female population in different periods in the U.S.

The variation trends of the age-specific mortality rates for cervical cancer among Chinese females aged 20–79 years during 1988–2012 are shown in Figure 2 and Figure 3. Similarly, the trends among U.S. females aged 20–79 years during 1953–2012 are shown in Figure 4. In general, the mortality rates for cervical cancer in urban China, rural China and the U.S. increased with age, regardless of the period effect. The mortality rates in urban China increased at a steady rate in the age groups younger than 55–59 years, and this trend was followed by a significant increase in mortality beginning in the 60 to 64-year-old age group that was observed during all time periods from 1988 to 2012. It’s worth noting that the mortality rates during 1988–1992 were higher than the rates observed during the other four periods and maintained the steepest increasing slope in the age groups including women aged 55 to 59 years and older. In rural China, the mortality rates increased significantly with age beginning in the age group including 40 to 44-year-old women, except for the period 2003–2007, which presented the lower mortality rates in the age group including 45 to 49-year-old women compared with the age group including 40 to 44-year-old women.

**Figure 5:**
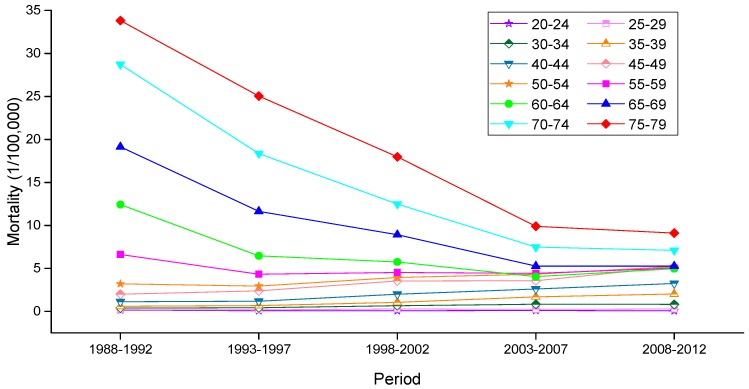
Mortality rates for cervical cancer per 100,000 female population of different age groups in urban China.

**Figure 6:**
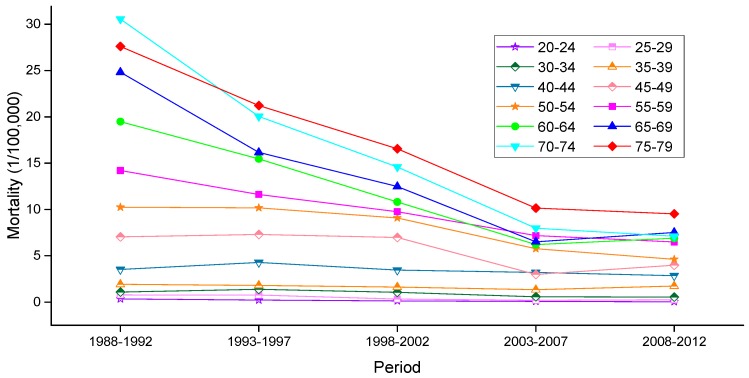
Mortality rates for cervical cancer per 100,000 female population of different age groups in rural China.

**Figure 7:**
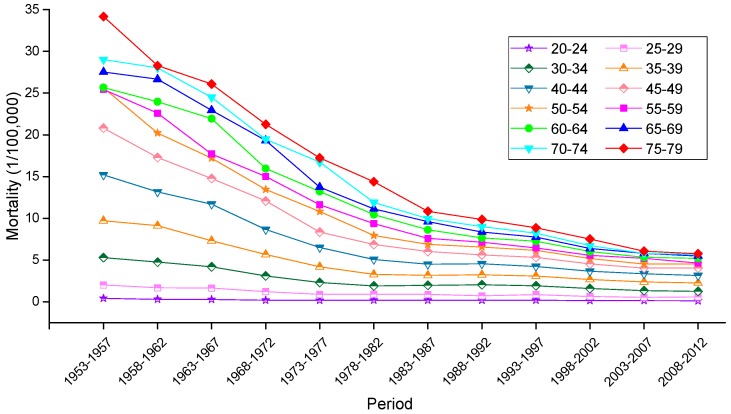
Mortality rates for cervical cancer per 100,000 female population of different age groups in the U.S.

Taken together, the mortality rates during the period 1988–1992 were higher than those observed during the other four periods, which was largely driven by the rate observed in the group aged 50–54 years; conversely, the cervical cancer mortality rates for all age groups during the periods 2003–2007 and 2008–2012 were relatively lower than those observed during the other three periods and showed the lowest rate of increase. In the United States, the mortality rates observed during different periods all presented an increasing trend with age, and the rates during the periods 1953–1957 and 2008–2012 demonstrated the highest and lowest levels in all age groups, respectively. The variation trends of the cervical cancer mortality rates in different age groups among Chinese and U.S. females are shown in Figure 5, Figure 6 and Figure 7. In urban China, the four groups including women aged 60–79 years clearly showed a sharp decreasing trend, while the age groups including women aged 30–49 years increased gradually, and the mortality rates observed in the remaining age groups fluctuated without a clear trend during the whole observation period. Overall, mortality increased over the entire observation period with older age group; this was particularly exhibited in the group of women aged 75–79 years, which declined at the fastest rate but maintained the highest level of mortality. Different from urban China, the trends in rural China indicated decreasing rates in all age groups during the whole period, except for the age group including women aged 45–49 years, which demonstrated an irregular fluctuating trend during 1988–2012. Additionally, mortality decreased more rapidly with increased age, while the mortality rates observed in older groups were higher than those in younger groups during the same period, indicating that the older groups were still at higher risk of death. 

In the United States, the mortality rates observed in different age groups generally exhibited a steady declining trend with time, except for the age groups containing women aged 30–34, 35–39 and 40–44 years, which demonstrated higher mortality rates during the period 1988–1992 than the period 1983–1987. Similar in trends to China, mortality in the U.S. increased with age during the entire observation period, and the mortality observed in the groups of women aged 60–79 years experienced a steeper decline with time period than did those of the other age groups. 

**Figure 8:**
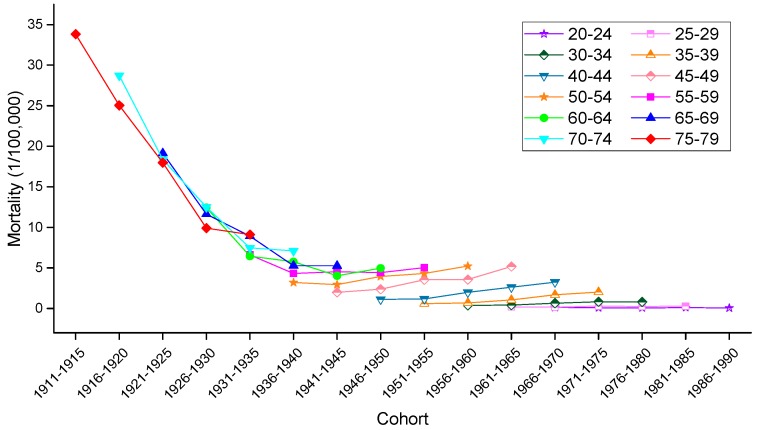
Mortality rates for cervical cancer per 100,000 female population by birth cohort in urban China.

**Figure 9:**
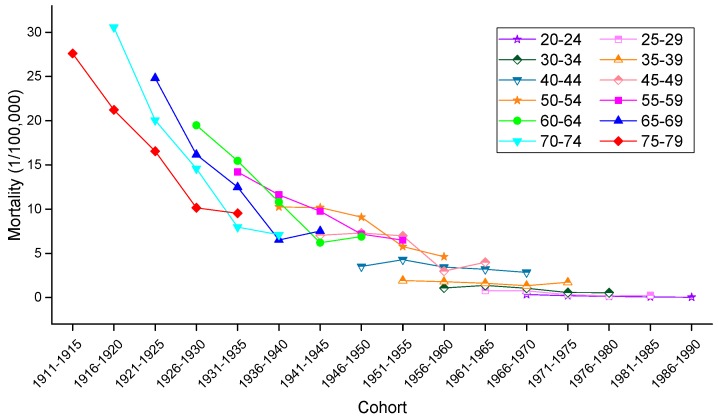
Mortality rates for cervical cancer per 100,000 female population by birth cohort in rural China.

**Figure 10:**
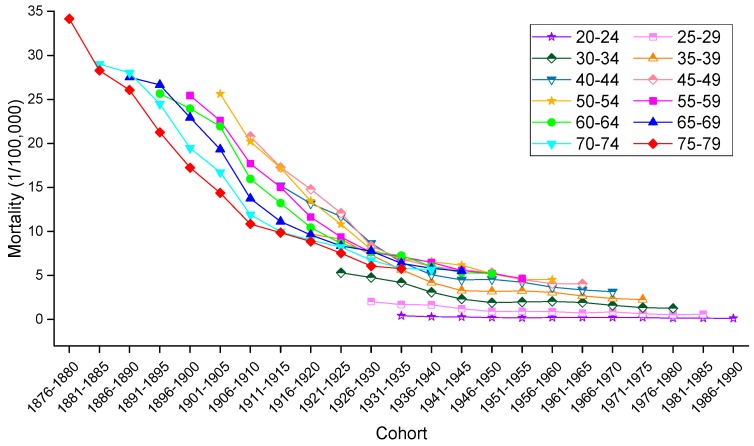
Mortality rates for cervical cancer per 100,000 female population by birth cohort in the U.S.

To allow for an intuitive evaluation of the effect of birth cohort on the variation in cervical mortality rates, the age-specific mortality rates observed in different birth cohorts in urban China, rural China and the U.S. are depicted in Figure 8, Figure 9 and Figure 10, respectively. Generally speaking, mortality rates fluctuated slightly in the younger age groups, and the older groups still suffered from a higher risk of mortality than did the younger groups. In urban China, the mortality rates observed in those born after 1946–1950 consistently increased with age but decreased in the 1916–1925 birth cohort, while the mortality rates observed in the 1926–1945 birth cohorts fluctuated erratically. Additionally, cancer mortality fluctuated unpredictably with changes in cohort year within the same age groups. In rural China, the mortality rates observed in the 20–24, 50–54, 55–59, 70–74 and 75–79 years age groups consistently decreased with cohort year, while this decrease was only observed in the other age groups to varying degrees. Similar increases with age were observed in the cohorts born in 1961–1965, 1966–1970, 1971–1975, 1976–1980 and 1981–1985, while the birth cohort including women born before the 1930s demonstrated a progressively decreasing trend with increased age.

Consistent with the overall trends, mortality rates in the United States fluctuated more violently in the older age groups than in the younger groups, which fluctuated gently over the whole cohort period. In particular, in a phenomenon noticeably different from that observed in China, the variation trend of the mortality rates was unevenly associated with age within the same birth cohort; nevertheless, the mortality rates observed in the cohorts born before 1945 consistently decreased within the same age groups, and the rates observed in the age groups including women aged 45–79 years declined steadily with cohort years. 

**Figure 11:**
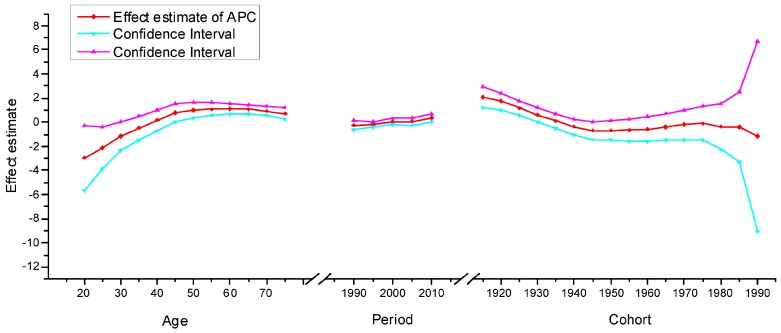
Age, period and cohort effects on cervical cancer mortality in urban China and the corresponding 95% confidence intervals.

**Figure 12:**
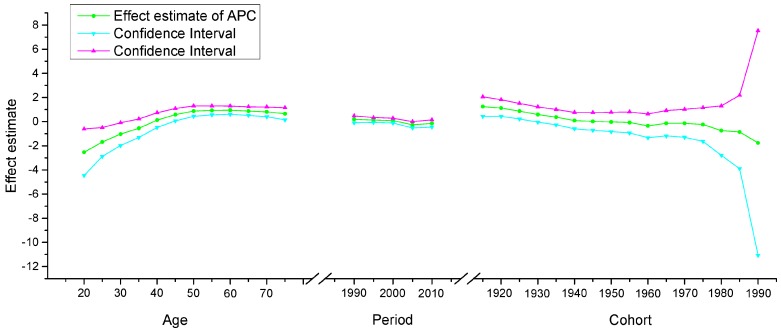
Age, period and cohort effects on cervical cancer mortality in rural China and the corresponding 95% confidence intervals.

**Figure 13:**
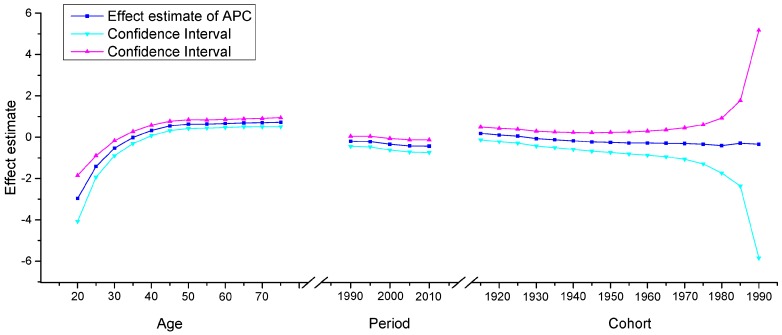
Age, period and cohort effects on cervical cancer mortality in the U.S. and the corresponding 95% confidence intervals.

The APC model was used to explore the age effect, period effect and cohort effect using the intrinsic estimator method. The results of the APC analysis of age-specific cervical cancer mortality are described in Table 1. Trends in the age effect, period effect and cohort effect in China and the U.S. are shown in Figure 11, Figure 12 and Figure 13. The separate effect of each element was investigated as follows:

*Age effect*. The age effect increased consistently with age in the age groups including women aged 20 to 64 years in all areas. In urban and rural China, the effect increased significantly before the age of 64 years and peaked in the age group including women aged 60–64 years. However, after the age of 64 years, age effects demonstrated a gradual decreasing instead of an increasing trend, which was different from the trends observed in the U.S. In contrast, the age effect in the U.S. consistently increased in all age groups from 20 to 79 years, particularly in the age groups including women aged 20–49 years, with rates increasing dramatically after the age of 20 years and plateauing after the age of 49 years. Generally speaking, the lowest coefficient of estimation for the age effect was observed in the group including women aged 20–24 years, which indicated that the group of patients aged 20–24 years had the lowest cervical cancer mortality risk.

*Period effect.* The period effect exhibited an obvious decline in rural China and the U.S. during the whole observation period, which indicated that the period effect contributed to the observed decrease in the linear trend of cervical cancer mortality, whereas an opposing trend was identified in urban China. Period effects declined steadily in both rural China and the U.S., while a slight increasing trend was identified (from −0.25 to 0.33) in urban China, which indicated an increasing mortality risk over time.

*Cohort effect.* The cohort effect showed differently fluctuating trends in the three areas. The effect in rural China and urban China presented similar variation trends in association with cohort year, which increased after the first decrease and then declined again, while the cohort effect in the U.S. generally declined steadily. Cohort effects peaked in the cohort born in 1911–1915 in both rural and urban China; consistently declined in the cohort born before 1950, and then decreased again in the cohort born after 1976–1980. The cohort effect in the U.S. peaked in the cohort born in 1876–1880, then leveled off and slightly decreased in younger generations. Overall, the mortality risk by birth cohort in China and the U.S. showed a declining trend except for during some periods, which demonstrated slightly increasing or decreasing trends.

## 4. Discussion

Cervical cancer is a type of malignancy affected by biological factors, genetic factors, personal behavior and other factors. The epidemiological characteristics of cervical cancer have been found to be associated with socioeconomic distribution, geographical position and population factors [27]. Human papillomavirus (HPV) infection is considered to be the most important risk factor among the multitudinous previously identified risk factors that include increased numbers of sexual partners, younger age at first sexual intercourse, smoking, oral contraceptive use and low socioeconomic status, according to relevant research [28].

To the best of our knowledge, APC analyses of cervical cancer mortality trends have already been successfully performed in many areas including Japan [29], Taiwan [2,14], Hong Kong [20] and the USA [30]. However, the performance of a systematic comparison of these trends in mainland China and the United States using the same analytical models appeared to be rare. Thus, we conducted this study, which focused on the comparison of cervical cancer mortality trends in urban China, rural China and the United States using age-period-cohort analysis methods to explore the cause of the cancer trends and assess the effect of public health control policies.

The overall trends observed in the three areas analyzed in our study suggested that the ASMR for cervical cancer in the United States decreased significantly during the period 1953–1982, but leveled off thereafter, which was generally consistent with the results of the study conducted by Vaccarella [28]. This significant decline was likely predominately related to the widespread implementation of Papanicolaou (Pap) cytology testing during the mid-1950s and introduction of the HPV vaccine. Vaccination against the human papilloma virus (HPV) can prevent infection with the most common serotypes identified as causing cervical cancer in the United States. However, the absorbed dose of vaccination has been found to vary in different populations in the United States [31,32]. Similarly, the ASMRs for cervical cancer declined gradually in both rural and urban China from 1988 to 2002, which likely reflected continuous improvement in living standards and health conditions. Furthermore, the ASMR trends in China demonstrated that beginning in 2005, cervical cancer mortality rates were higher in urban than rural women, and this situation urgently requires the implementation of effective measures to reduce the mortality rate in urban women.

In general, effects of age on cervical cancer were affected by biological factors, and overall mortality increased with age and decreased with birth cohort. For patients in urban China and rural China, the age effects revealed in the APC analysis were generally observed to increase significantly before the 64 years, while the age effect consistently increased until and then plateaued after the age of 49 years in the United States. A significantly increasing rate was observed in women younger than 49 years old in the United States, which indicated that the risk of cervical cancer tended to increase in the middle-aged and younger groups. This finding was, for the most part, consistent with the research conducted by Benard [33]. The increasing trend observed in the age effect in the U.S. may suggest that as age increased, the risk of mortality increased, which could be explained by the fact that older women may experience greater exposure to low hygiene environments and have less opportunities to engage with preventive programs than those in younger age groups. In contrast, a slightly decreasing trend was observed in women older than 64 years old in rural and urban China, which indicated that cervical cancer mortality was relatively low in the elderly and that cervical cancer deaths tended to occurred in the younger age groups. This phenomenon may be possibly explained by the influence of female sex hormones, the assessment of small numbers of elderly individuals and incomplete reporting from institutions [14]. In fact, this finding was similar to that observed in the study conducted by Zhao [34].

**Figure 14:**
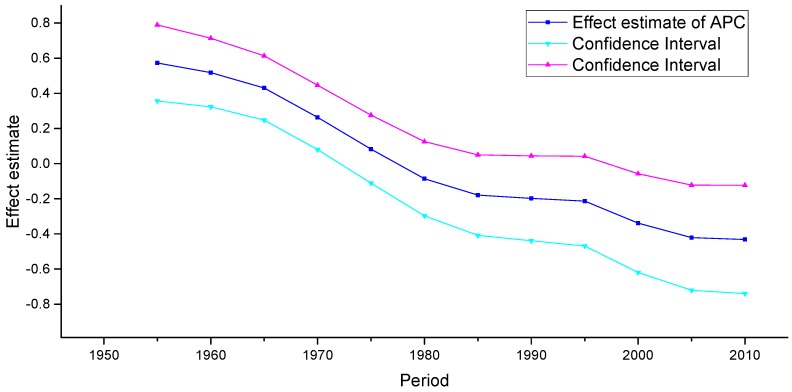
Period effect on cervical cancer mortality from the results of APC-IE analysis in the U.S.

Period effects reflected factors with immediate effects on cancer mortality and incidence, such as development of effective treatments and implementation of screening [35]. For the three areas analyzed in our study, small period effects were observed over the whole study periods. According to detailed analysis of the effect of period of cervical cancer mortality, we observed conflicting trends in the three areas. Similar decreasing trends were discovered over the whole observation period in rural China and the U.S., which indicated that the period effect contributed to the decrease in the linear trend of cervical cancer mortality. In contrast, an increasing trend was observed in urban China, which was generally consistent with the increasing linear trend of cervical cancer mortality observed beginning in 2002. This phenomenon, however, might indicate that these period effects were associated with increased risk of cervical cancer mortality in urban China, indicating that period might be an important factor affecting trends in cancer mortality. Although a reduction in cervical cancer mortality risk was expected to be observed due to the improvement of medical conditions and implementation of cytology screening that have occurred since the 1960s, increasing period effects were still observed, which were likely affected by environmental factors and dietary patterns. It should be noted that because the study period evaluated in China (1988–2012) was relatively short, we could not specifically analyze the effect of period; thus, long-term data from the United States from 1953 to 2012 was analyzed using the APC model to explore the period effect more accurately. The trends in the period effect in the United States are shown in Figure 14. According to Figure 14, the period effect in the United States began to decrease in 1953 and plateaued after 1982, and the ASMR in the United States, as presented in Figure 1, fluctuated simultaneously. Accordingly, we concluded that although the effect of period was observed to be small, it might be the key factor affecting the trends in cervical cancer mortality through the implementation of clinical treatment and screening program.

Generally speaking, the cohort effect reflected the impact of different risk factors for cervical cancer such as HPV infection, HSV-2 infection, number of sexual partners, younger age at first sexual intercourse, smoking and oral contraceptive use in different generations [28], and the mortality risk associated with birth cohorts in China and the U.S. showed a general declining trend. The effect of cohort declined steadily in the United States and peaked in the cohort born in 1876–1880, which indicated a decreased risk of mortality in younger generations. However, a slight upward trend was observed in the cohorts born after 1980, which may possibly be explained by earlier onset of sexual activity and the opportunistic cervical cancer screening performed during gynecological examinations in the United States [35]. Fluctuating trends in cohort effects were observed in urban and rural China, and decreased mortality rate were identified in the cohorts born in 1911–1950 and 1976–1990. In urban and rural China, the decreased cohort effects observed in younger generations were probably due to a decrease in the prevalence of cancer risk factors related to the improvements in public health policy, treatment of medical conditions and implementation of cervical cancer screening programs that have occurred in recent years. However, the increased cohort effect observed in those born during 1945–1975 in urban China may be explained by earlier onset of sexual activity, which also simultaneously increased the prevalence of HPV.

In the United States, cervical cancer mortality has been decreasing at a consistent rate across most states; this pattern, nevertheless, was different from that identified in China. To achieve the target of reducing cervical mortality, intensive efforts have been made, and free or low-cost cervical cancer screening and health services have been provided for more than 20 years [5]. However, the absence of a national cervical cancer screening program and a lack of awareness about the disease resulted in a slight increase in cervical mortality during a period of time in China. Here, it should be noted that exposure to risk factors increased following rapid economic development and changes in patterns of individual behavior. Thus, when cervical cancer screening guidelines are developed in the future, risk factors affecting different socioeconomic populations should be considered. To better prevent and treat this disease, regular and effective free screening should be widely performed in the different regions of China [7].

In China, cervical cancer mortality in female populations was previously higher in rural China than in urban China, which was likely due to the relatively backward economy and a lack of awareness about disease. However, this trend reversed beginning in the year 2005, which may have been driven by urbanization, reform and rapid economic transitions. This phenomenon suggested that the Chinese government should pay more attention to the targeting of cancer screening towards the high‑risk populations. Fortunately, however, an overall decreasing trend in cervical cancer mortality in China was observed during the study period, which was likely due to improvements in living standards and medical care; meanwhile, the development of the HPV vaccine and the implementation of screening programs promoting primary and secondary prevention of cervical cancer should be the focus of prevention and control work in the future.

Some specific limitations exist in our study. A major limitation is that the incidence of cervical cancer in China and the United States was not analyzed, and incidence plays a critical role in the development of cervical cancer. Thus, further analysis of cervical cancer incidence needs to be performed in the future. Moreover, the sources of the data for the United States and China were only partially the same during the years from 1987 to 2000, which limited the comparability of the information obtained from these databases. Additionally, the observation period in China was relatively short (from 1988 to 2012), and data in 2001 for urban and rural China was missing, which may also affect the representativeness and comprehensiveness of our results. However, we also collected mortality data for the United States to explore the long-term period effect from 1953 to 2012, which helped us to analyze secular trends more accurately to a certain extent. Furthermore, despite the non-bias, validity, asymptotic features and superior estimation ability of the IE method, the parameter estimates generated using this method are not intuitive, which limits their extensive application. Moreover, the theory behind this method is complicated; therefore, further studies are still needed to explain the actual meaning of parameter estimates in the future.

## 5. Conclusions

In summary, we evaluated the general trends in cervical cancer mortality rates in China and the United States during the periods 1988–2012 and 1953–2012, respectively. Overall, declining trends were observed in the two countries before 2002, while an increasing trend was observed in urban China after 2002, and the trend in rural China fluctuated significantly over the observation period. Generally speaking, mortality rates demonstrated a decreasing trend in both countries; however, the declining trend in the U.S. was relatively stable compared to the trend in China, which may be attributed to widespread implementation of Papanicolaou (Pap) cytology testing in the mid-1950s and introduction of the HPV vaccine in the U.S. Moreover, a re-ascending mortality trend in China has been observed in recent years, which might indicate that the implementation of public health control policies has been inadequate, and limited medical resources and clinical treatment may have driven the increasing mortality rates observed in the Chinese female population. Our study shows that in general, cervical cancer mortality rates in females increased with age and decreased with birth cohort in the U.S., while the risk of mortality increased with birth cohort in those born during 1946–1975 in urban China. Additionally, the risk of mortality increased continuously with age before the age of 64 years in urban and rural China and began to decline in older age groups. Although the age and cohort effects were relatively strong, the period effect may be the key factor affecting trends in cervical cancer mortality, mainly reflecting the immediate effects of effective treatment and screening implementation.

## Figures and Tables

**Table 1:** APC model analysis results of cervix uteri cancer mortality.

Mortality	Urban China		Rural China		U.S.	
	Coef.	SE	Coef.	SE	Coef.	SE
Age (year)						
20–24	−2.95	1.38	−2.53	0.99	−2.96	0.57
25–29	−2.13	0.86	−1.69	0.61	−1.41	0.27
30–34	−1.17	0.62	−1.04	0.48	−0.53	0.19
35–39	−0.48	0.50	−0.54	0.39	−0.01	0.15
40–44	0.16	0.43	0.13	0.31	0.33	0.13
45–49	0.78	0.36	0.58	0.26	0.54	0.11
50–54	0.97	0.32	0.87	0.22	0.63	0.11
55–59	1.07	0.27	0.93	0.19	0.64	0.10
60–64	1.08	0.22	0.95	0.17	0.66	0.10
65–69	1.05	0.20	0.87	0.18	0.69	0.10
70–74	0.92	0.20	0.81	0.21	0.70	0.10
75–79	0.70	0.26	0.65	0.26	0.72	0.11
Period (year)						
1990	−0.25	0.19	0.19	0.14	−0.20	0.12
1995	−0.20	0.13	0.13	0.11	−0.21	0.13
2000	0.06	0.12	0.09	0.10	−0.34	0.14
2005	0.07	0.16	−0.26	0.13	−0.42	0.15
2010	0.33	0.18	−0.15	0.15	−0.43	0.16
Cohort (year)						
1911–1915	2.09	0.44	1.25	0.41	0.18	0.16
1916–1920	1.72	0.35	1.13	0.35	0.11	0.17
1921–1925	1.18	0.31	0.87	0.33	0.05	0.17
1926–1930	0.61	0.29	0.59	0.32	−0.07	0.18
1931–1935	0.09	0.29	0.37	0.32	−0.13	0.19
1936–1940	−0.38	0.33	0.09	0.35	−0.18	0.21
1941–1945	−0.69	0.38	0.02	0.37	−0.23	0.23
1946–1950	−0.70	0.42	−0.03	0.41	−0.25	0.25
1951–1955	−0.66	0.47	−0.07	0.45	−0.28	0.27
1956–1960	−0.58	0.52	−0.35	0.50	−0.28	0.30
1961–1965	−0.39	0.57	−0.13	0.54	−0.29	0.33
1966–1970	−0.22	0.63	−0.13	0.59	−0.31	0.39
1971–1975	−0.12	0.72	−0.24	0.71	−0.34	0.49
1976–1980	−0.38	0.97	−0.74	1.04	−0.40	0.68
1981–1985	−0.41	1.47	−0.86	1.55	−0.30	1.05
1986–1990	−1.17	4.02	−1.76	4.75	−0.34	2.81
Deviance	1.15		2.58		1.19	
AIC	3.87		4.26		4.13	
BIC	−121.68		−120.25		−495.79	

## Appendix A

**Table A1:** Age-specific mortality rates (per 100,000 women) of cervix uteri cancer in urban China.

Age	Year of Death				
	1988–1992	1993–1997	1998–2002	2003–2007	2008–2012
20–24	0.14	0.08	0.07	0.10	0.06
25–29	0.20	0.18	0.29	0.24	0.29
30–34	0.38	0.42	0.66	0.83	0.81
35–39	0.60	0.68	1.05	1.69	2.02
40–44	1.12	1.18	2.00	2.61	3.25
45–49	2.00	2.38	3.54	3.57	5.18
50–54	3.20	2.94	3.94	4.32	5.23
55–59	6.62	4.32	4.53	4.43	5.03
60–64	12.42	6.46	5.75	4.04	4.96
65–69	19.14	11.64	8.92	5.27	5.26
70–74	28.72	18.36	12.49	7.48	7.10
75–79	33.82	25.04	17.98	9.90	9.11

**Table A2:** Age-specific mortality rates (per 100,000 women) of cervix uteri cancer in rural China.

Age	Year of Death				
	1988–1992	1993–1997	1998–2002	2003–2007	2008–2012
20–24	0.34	0.22	0.13	0.07	0.04
25–29	0.78	0.76	0.33	0.19	0.25
30–34	1.08	1.38	1.05	0.58	0.55
35–39	1.92	1.80	1.62	1.34	1.73
40–44	3.52	4.28	3.45	3.20	2.85
45–49	7.04	7.30	6.98	2.99	3.99
50–54	10.24	10.18	9.09	5.76	4.62
55–59	14.20	11.62	9.76	7.18	6.49
60–64	19.48	15.46	10.81	6.21	6.89
65–69	24.82	16.16	12.48	6.51	7.52
70–74	30.56	20.04	14.58	7.97	7.11
75–79	27.60	21.22	16.55	10.15	9.53

**Table A3:** Age-specific mortality rates (per 100,000 women) of cervix uteri cancer in U.S.

Age	Year of Death											
	1953–1957	1958–1962	1963–1967	1968–1972	1973–1977	1978–1982	1983–1987	1988–1992	1993–1997	1998–2002	2003–2007	2008–2012
20–24	0.42	0.30	0.28	0.20	0.18	0.20	0.20	0.20	0.20	0.14	0.14	0.12
25–29	2.02	1.68	1.64	1.22	0.90	0.92	0.88	0.72	0.86	0.64	0.54	0.58
30–34	5.30	4.76	4.20	3.10	2.32	1.90	1.98	2.04	1.92	1.60	1.34	1.26
35–39	9.70	9.12	7.32	5.66	4.20	3.28	3.18	3.24	3.08	2.68	2.38	2.26
40–44	15.20	13.16	11.70	8.66	6.50	5.08	4.50	4.56	4.24	3.66	3.36	3.14
45–49	20.80	17.28	14.78	12.08	8.36	6.86	6.02	5.62	5.32	4.54	4.04	4.04
50–54	25.62	20.22	17.20	13.46	10.82	7.94	6.88	6.56	6.16	5.18	4.52	4.52
55–59	25.44	22.58	17.70	15.02	11.62	9.36	7.60	7.14	6.46	5.56	5.20	4.64
60–64	25.66	23.96	21.94	15.96	13.22	10.44	8.62	7.64	7.26	6.02	5.32	5.20
65–69	27.52	26.66	22.94	19.32	13.74	11.12	9.60	8.36	7.76	6.38	5.80	5.48
70–74	29.00	28.02	24.50	19.46	16.72	11.90	9.96	9.00	8.22	6.72	5.80	5.66
75–79	34.16	28.28	26.08	21.26	17.24	14.38	10.84	9.86	8.86	7.52	6.06	5.76
